# The Balance between Conventional DCs and Plasmacytoid DCs Is Pivotal for Immunological Tolerance during Pregnancy in the Mouse

**DOI:** 10.1038/srep26984

**Published:** 2016-05-27

**Authors:** Wen-ning Fang, Meng Shi, Chao-yang Meng, Dan-dan Li, Jing-pian Peng

**Affiliations:** 1 State Key Laboratory of Stem Cell and Reproductive Biology, Institute of Zoology, Chinese Academy of Sciences, Beijing, People’s Republic of China; 2 University of Chinese Academy of Sciences, Beijing, People’s Republic of China; 3 School of Biological Sciences, Nanyang Technological University, Singapore

## Abstract

Dendritic cells (DCs), which can shape their functions depending on the microenvironment, are crucial for the delicate balance of immunity and tolerance during pregnancy. However, the mechanism underlying the microenvironment-educated plasticity of DC differentiation during pregnancy remains largely unclear. Here, we found that the differentiation of conventional DCs (cDCs) and plasmacytoid DCs (pDCs) is regulated in a tissue-specific manner during pregnancy. The ratio of cDCs and pDCs remained constant in the spleen. However, the ratio changed in the para-aortic lymph nodes (LNs), where cDC percentages were significantly reduced concurrent with an increase in pDCs from E8.5 to E16.5. Moreover, the expansion of pDCs and T regulatory (Treg) cells was correlated in the para-aortic LNs, and pDCs had more potential to induce regulatory T cells (Tregs) compared with cDCs (independent of IDO expression). Notably, the balance between cDCs and pDCs is disrupted in IFN-γ-induced abnormal pregnancy, accompanied by lower Treg percentages in the para-aortic LNs and decidua. To further identify the underlying mechanism, we found that elevated IFN-γ can increase the levels of GM-CSF to alter the differentiation of pDCs into cDCs *in vivo*. Therefore, we provide a novel regulatory mechanism underlying pregnancy-related immune tolerance that involves the balance of DC subsets, which may offer a new target for the prevention of human spontaneous abortion.

Successful pregnancy not only establishes immune tolerance for a semiallogeneic fetus but also maintains adequate defense against pathogens. In this process, DCs play a critical role in immunomodulation by balancing tolerance and immune responses^1^. On one hand, the retention of intrinsic immunogenicity by uterine DCs (uDCs) is a potential threat to fetal survival because these cells present fetal/placental antigens to activate T cells in uterine-draining lymph nodes (uLNs)^2^. To minimize this risk, uDCs, the densities of which are dramatically reduced in the decidua^3^, are also trapped in sites and unable to migrate to activate immune responses in uLNs^2^. Additionally, the diminished immunogenic ability of uDCs is due to their appropriate maturation state during normal pregnancy, whereas a more mature phenotype is observed in a highly abortion-prone CBA/J X DBA/2J murine model^4^. On the other hand, uDCs generate significantly higher levels of IL10 than IL12^5^, which may be crucial for promoting tolerance by inducing Treg cells^6^ during gestation. Nevertheless, the tolerant state of DCs can be disrupted by some inflammatory factors, leading to pregnancy failure. Although several mechanisms underlying this immune tolerance have been revealed, it remains far from fully understood. Many studies have characterized uDCs as a “CD11c^+^” population; however, they have fairly high heterogeneity^7^, existing as a plethora of DC subsets with various functions. Furthermore, the composition of different DC subsets also varies considerably in response to normal or pathological microenvironment^4^. Therefore, exploring the plasticity of special DC subsets in normal and abnormal pregnancy will deepen our understanding of maternal-fetal immune tolerance.

In humans, two distinct lineages of DC subsets, myeloid DCs (mDCs) and plasmacytoid DCs (pDCs), have been identified in the first-trimester decidua^8^. The frequency of mDCs was significantly higher in the serum of patients suffering from preeclampsia, while pDC levels were lower compared with the control^9^, suggesting that the balance between mDCs and pDCs may be important for maintaining normal pregnancy. In addition, the function of pDCs during pregnancy remains poorly understood. PDCs have been confirmed to have multifaceted biological functions to facilitate immunomodulation in innate and adaptive immunity. One prominent characteristic of pDCs is to produce high quantities of type I interferons (IFNs) immediately upon recognizing viruses or self nucleic acids via Toll-like receptor 7 (TLR7) and TLR9 to contribute to innate antiviral immunity^10^,^11^. Additionally, pDCs can present antigens to T cells because they express MHC class II and certain co-stimulatory molecules, such as CD40, CD80 and CD86, although their presenting efficiency is lower than that of cDCs^12^. Furthermore, pDCs can activate or inhibit immunoreactions in a microenvironment-dependent manner; they can activate CD4^+^ T cells by receiving signals from TLRs or other pattern recognition receptors, whereas they promote tolerance when they are unstimulated or alternatively activated. For instance, because they are tolerogenic rather than immunogenic, elevated numbers of pDCs in tumors are associated with a poor prognosis, and alternatively activated tumor-infiltrating pDCs can induce Tregs via the expression of indoleamine 2, 3-dioxygenase (IDO)^13^,^14^,^15^,^16^ or ICOSL^17^. Additionally, TGF-β and TNF generated by tumor cells^18^ can suppress type I IFN secretion^19^,^20^ and reduce the immunogenic capacity of pDCs. The acceptance of a semiallogeneic fetus during pregnancy requires a similar type of immunomodulation to that of a tumor escaping from host immunosurveillance^21^; however, the role of pDCs in peripheral and maternal-fetal tolerance is unknown.

Fms-like tyrosine 3 (Flt3) signaling is crucial for pDC development^22^, and pDCs in both lymphoid organs and bone marrow are decreased when this signaling is absent^23^. Flt3 ligand (Flt3L) promotes pDC development through activating STAT3^24^ and mTOR^25^ and can also cooperate with type I IFNs to expand pDCs^26^. In contrast, GM-CSF inhibits FLT3L-induced pDC differentiation^27^ by activating STAT5 signaling to downregulate IRF8, which is indispensable for pDC development^28^. IFN-γ was found to be significantly elevated in patients with preeclampsia^29^ or unexplainable recurrent spontaneous abortion^30^, and a supraphysiological level of IFN-γ can have deleterious effects, leading to pregnancy failure in mice^31^. Nevertheless, the correlation between an imbalance of Th1/Th2 cytokines and the differentiation of DC subsets during pregnancy failure remains elusive. IFN-γ-induced abortion in mice is an ideal model to evaluate the plasticity of DC subsets for different pregnancy outcomes.

In this study, we demonstrate that the proportion of cDCs and pDCs is precisely regulated during pregnancy, and their balance can be disrupted along with a reduction in Treg cells in IFN-γ-induced abnormal pregnancy with fetal abortion through enhanced GM-CSF expression. This is the first evidence indicating that the balance between cDCs and pDCs is an important immunoregulatory factor for successful pregnancy.

## Results

### Expansion of pDCs and Treg cells is correlated in the para-aortic LNs

**Figure 1:**
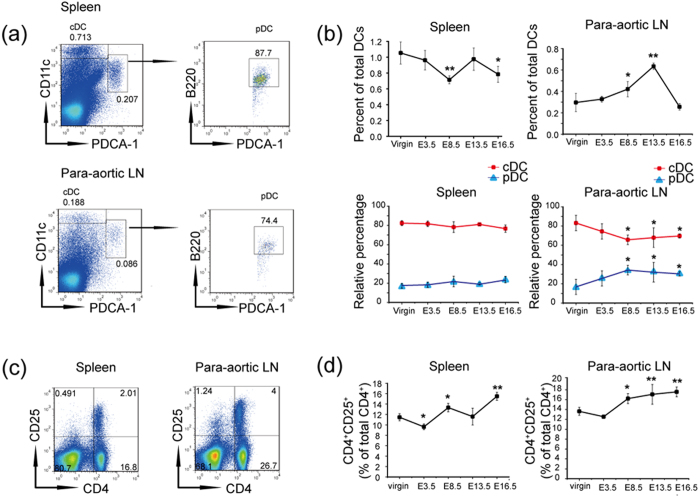
The expansion of Treg cells is highly correlated with pDCs but inversely correlated with cDCs in the para-aortic LNs during pregnancy. Virgin mice and allogeneic mated female C57BL/6 mice were sacrificed on E3.5, E8.5, E13.5, and E16.5. Cell suspensions were prepared from the detached tissues for analysis by flow cytometry. (**a**) Boxes depict the gating strategy for cDCs and pDCs in the spleens and para-aortic LNs of virgin mice as an example. (**b**) Analysis of the frequency of total DCs in the leukocytes and the relative percentages of cDCs and pDCs in the total DCs at different time points of gestation. Non-pregnant mice served as a control. The data show the mean ± SD and were obtained from at least three mice. *P < 0.05; **P < 0.01. (**c**) Boxes depict the gating strategy for CD4^+^ CD25^+^ T cells in the spleens and para-aortic LNs of virgin mice as an example. (**d**) Percentages of CD4^+^ CD25^+^ T cells in the leukocytes of non-pregnant and gravid mice. The data show the mean ± SD and were obtained from at least three mice. *P < 0.05; **P < 0.01.

The proportions of mDCs and pDCs are altered in patients with preeclampsia^9^, but the adverse effects of this change for normal pregnancy remain unknown. To explore this question, we first examined the percentages of mDCs and pDCs at different stages of pregnancy. Human mDCs are equivalent to conventional DCs (cDCs), a CD11c^+^ population in the mouse, and mouse pDCs are characterized by the surface expression of CD11c^− to lo^B220^+^ PDCA-1^+^, as described previously^32^. As shown in Fig. 1a, the two DC subsets gated in the spleens and para-aortic LNs of non-pregnant mice were used as a control, and the cDCs and pDCs of gravid mice were analyzed via flow cytometry on successive gestational days, such as E3.5, E8.5, E13.5, E16.5, and compared with those from virgin mice (Fig. 1b). The total DCs (the sum of cDCs and pDCs) in the spleens were downregulated significantly on E8.5, while the relative percentages of cDCs and pDCs remained constant at different gestational stages. However, the total DC percentages were elevated significantly from E8.5 to E13.5 and then decreased until E16.5 in the para-aortic LNs, wherein the proportion of cDCs was reduced from E8.5 to E16.5 compared with virgin mice and was accompanied by an increase in pDCs from E8.5 to E16.5. In detail, the cDC percentage reached their lowest level on E8.5, concomitant with the highest pDC percentage. These results suggest that the proportion change in the DC subsets may be a key factor in immunomodulation in the uLNs. Next, because pDCs can mediate tolerance by inducing the development of alloantigen-specific Treg cells in the LNs^33^, we evaluated the frequency of CD4^+^ CD25^+^ Treg cells in the spleens and para-aortic LNs at the same time points. As shown in Fig. 1c,d, the expansion of Treg cells correlates with that of pDCs in the para-aortic LNs, but is inversely correlated with cDCs. In the spleens, there is no obvious correlation between the expansion of Treg cells and the two DC subsets.

**Figure 2:**
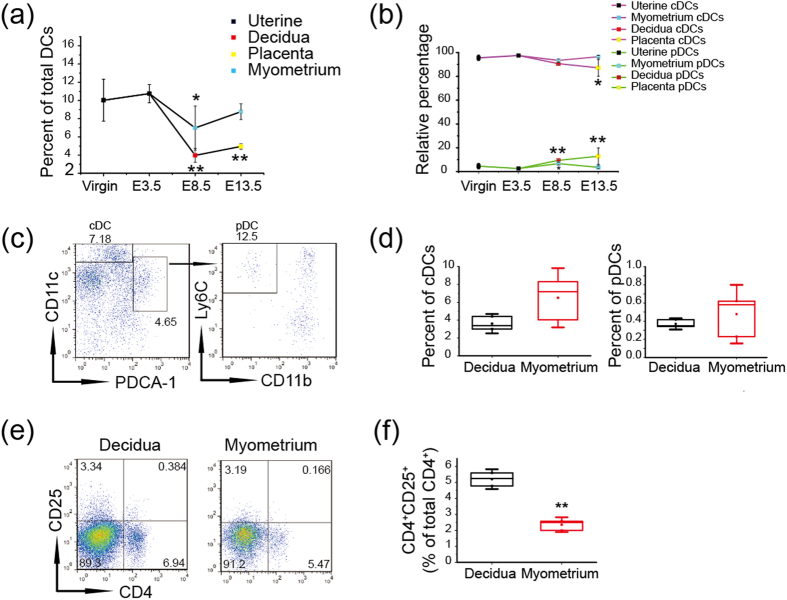
There is no correlation between the expansion of pDCs and CD4^+^ CD25^+^ T cells in the decidua. Virgin mice and allogeneic mated female C57BL/6 mice were sacrificed on E3.5, E8.5, E13.5 respectively, and cell suspensions were prepared from the separated tissues. The percentages of DC subsets and CD4^+^ CD25^+^ T cells were assessed in viable CD45^+^ leukocytes using flow cytometry. Analysis of the frequency of total DCs (**a**) and the relative percentages of cDCs and pDCs (**b**) at different time points of gestation (**c**) Boxes depict the gating strategy for cDCs and pDCs in the myometrium of gravid mice as an example. (**d**) Histogram displays the statistical results of the percentages of cDCs and pDCs in the decidua and myometrium of three mice. (**e**,**f** ) Assessing the percentage of CD4^+^ CD25^+^ T cells in viable leukocytes from the decidua and myometrium of three mice. The data show the mean ± SD. *P < 0.05; **P < 0.01.

PDCs have been rarely found in the human decidua during early pregnancy^8^, and different regulatory mechanisms underlying DC differentiation^3^ and migration^2^ exist between the decidua and myometrium in mice. Therefore, we analyzed the percentages of cDCs and pDCs from uterus of virgin and gravid mice at E3.5, the decidua and myometrium on E8.5 as well as placenta and myometrium on E13.5, respectively. Here, pDCs were characterized as CD11c^− to lo^PDCA-1^+^ CD11b^lo^Ly6C^+^ cells in the uterus, and the percentages of total DCs in the leukocytes from the deciduas on E8.5 and from the placenta on E13.5 were downregulated significantly, wherein the proportions of pDCs were increased in the deciduas on E8.5 and in the placenta on E13.5 compared with that of virgin mice, respectively (Fig. 2a,b). Furthermore, we quantified the proportions of cDCs and pDCs in the decidua and myometrium on E8.5 to evaluate their relationship with Treg cells. The proportions of cDCs and pDCs were both higher in the myometrium relative to the decidua, with no significant difference (Fig. 2c,d), while lower levels of CD4^+^ CD25^+^ T cells were observed (Fig. 2e,f). Thus, the data indicate that pDCs may not be the decisive factor in inducing elevated proportion of Treg cells in the decidua.

### PDCs have more potential to induce Treg cells independent of IDO expression

**Figure 3:**
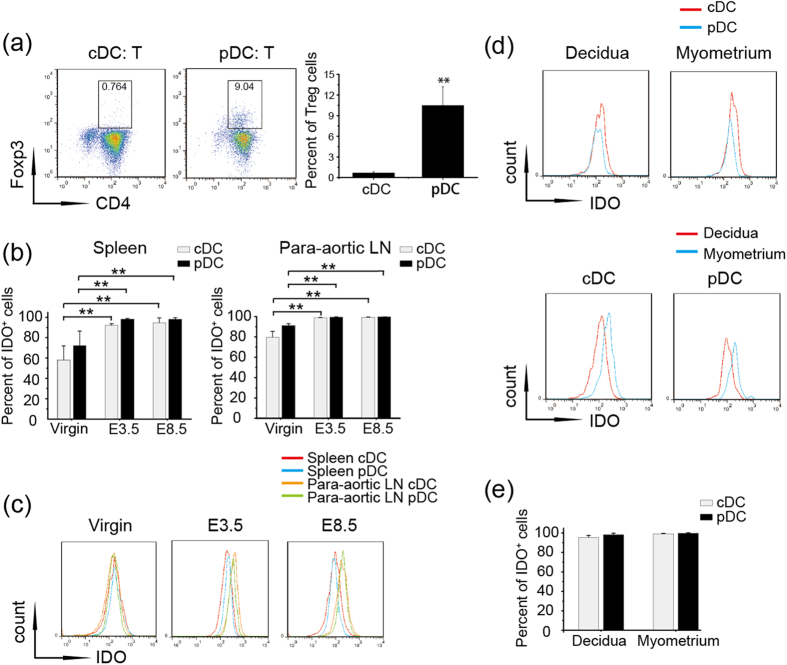
PDCs are more effective than cDCs at Treg induction independent of IDO expression. (**a**) CDCs and pDCs were sorted from the para-aortic LNs of gravid mice, and CD4^+^ CD25^−^ T cells were sorted from the spleens of OTII mice. Different DC subsets were co-cultured with CD4^+^ CD25^−^ T cells for 7 days, and CD4^+^ CD25^+^ FoxP3^+^ Treg cells were detected by flow cytometry. *Left panel*-Representative FACS profiles are shown; *Right panel*- Histogram displays the statistical results of three independent experiments. (**b**) Virgin mice and allogeneic mated female C57BL/6 mice were sacrificed on E3.5 or E8.5, and IDO^+^ populations of cDCs and pDCs from the spleens and para-aortic LNs of four mice were analyzed. (**c**) Representative flow cytometry depicting MFI values for IDO expression in the spleens and para-aortic LNs on the indicated days from three independent experiments. (**d**,**e**) Cell suspensions were prepared from the separated decidua and myometrium of gravid mice at E8.5. The data summarize the percentages of IDO^+^ cDCs and pDCs (**d**) and the MFI value of IDO in the two subsets in the decidua and myometrium (**e**). The data represent the mean ± SD of three independent experiments from at least three mice. *P < 0.05; **P < 0.01.

Because the expansion of pDCs and Treg cells is highly correlated in the para-aortic LNs, we further evaluated the Treg-inducing ability of cDCs and pDCs that were aseptically isolated from the para-aortic LNs of female C57BL/6 gravid mice and co-cultured with CD4^+^ CD25^−^ T cells from the spleens of OTII mice. The percentages of Treg cells, detected on D7, demonstrated that pDCs had more intrinsic potential to induce Treg cells (Fig. 3a).

Tolerogenic pDCs tend to induce Treg cells by promoting IDO expression^15^. Therefore, we determined whether the different capacities of cDCs and pDCs for Treg induction were due to a discrepancy in IDO expression. First, we gathered cells from the spleens and para-aortic LNs of virgin mice or pregnant mice on E3.5 and E8.5 to detect the IDO^+^ population within the cDCs and pDCs. Notably, the IDO-positive population was expanded significantly in both cDCs and pDCs during pregnancy progression, while no difference was found in the proportion of the IDO^+^ population between the spleens and para-aortic LNs (Fig. 3b). Next, we measured the fluorescence intensity, indicating the IDO expression level, which was elevated to a similar extent in both cDCs and pDCs from the para-aortic LNs compared with that those from spleens on E3.5 or E8.5, except for in virgin mice (Fig. 3c). These results suggest that pDCs have more potential to intrinsically induce the differentiation of Treg cells independent of the level of IDO expression.

Next, we compared the percentages of IDO^+^ cells and the fluorescence intensity of IDO in cDCs and pDCs from the decidua and myometrium on E8.5. As shown in Fig. 3d,f, little difference existed in the frequency of the IDO^+^ population and fluorescence intensity of IDO between the two DC subsets; notably, cDCs and pDCs exhibited stronger IDO fluorescence intensity in the myometrium. These data further confirm the role of pDCs in inducing Treg cells in the decidua independent of IDO expression.

### The balance between cDCs and pDCs is disrupted in IFN-γ-induced abnormal pregnancy

**Figure 4:**
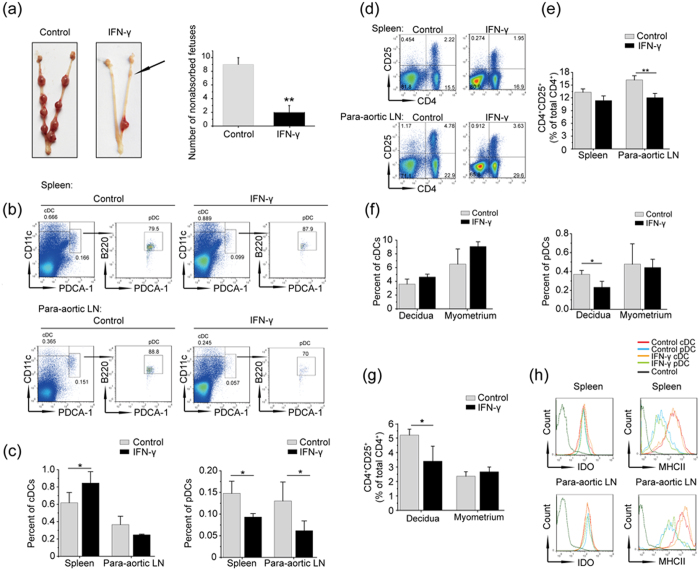
The balance of cDCs and pDCs is disrupted in IFN-γ-induced fetal resorption mice. Allogeneic mated C57BL/6 mice were intraperitoneally injected with solvent or IFN-γ for 3 continuous days from E5.5 to E7.5 and killed on E8.5. (**a**) Representative pictures show the healthy and aborted uteruses following solvent or IFN-γ treatment. *Right panel*-Histogram displays the statistical results. (**b**) Cell suspensions were prepared from the spleens and para-aortic LNs of normal and aborted mice. The boxes depict the gates and the percentages of the cDC and pDC populations. (**c**) Histogram displays the statistical results. (**d**) The percentages of CD4^+^ CD25^+^ T cells in the spleens and para-aortic LNs from solvent- or IFN-γ-treated mice; the statistical analyses are shown (**e**). The data represents the mean ± SD of three independent experiments and was obtained from at least three mice per group. *P < 0.05; **P < 0.01. The data represent the mean ± SD. *P < 0.05; **P < 0.01. (**f**,**g**) The decidua and myometrium of two implantation sites separated from a normal pregnancy mouse were taken as control, whilst the decidua and myometrium separated from one or two IFN-γ-treated mice were pooled for using in an independent experiment. Cell suspensions were prepared using the decidua and myometrium from normal and aborted mice on E8.5. The percentages of cDCs, pDCs (**f**) and CD4^+^ CD25^+^ T cells (**g**) were analyzed. The data represents the mean ± SD of three independent experiments. *P < 0.05; **P < 0.01. (**h**) Representative FACS profile shows the MFI values of IDO and MHCII in the cDCs and pDCs from the spleens and para-aortic LNs of normal and aborted mice in three independent experiments.

Up-regulated IFN-γ levels in the peripheral blood have been found in pregnant women with recurrent spontaneous abortion^30^, and ultra-physiological doses of IFN-γ have been demonstrated to result in pregnancy failure in mice^31^. Therefore, we confirmed whether the development of cDCs and pDCs can be altered by elevated IFN-γ. First, an IFN-γ-induced abnormal pregnancy model was established by injecting mice with IFN-γ intraperitoneally for 3 continuous days, and noticeable fetal absorptions were observed as abnormal pregnancies (Fig. 4a).

Then, cell suspensions were prepared from the spleens and para-aortic LNs of solvent- or IFN-γ-injected mice to evaluate cDCs and pDCs by flow cytometry. As shown in Fig. 4b,c, the proportions of cDCs in the spleens were significantly higher, and the levels of pDCs were lower in IFN-γ-injected mice relative to the control group. In the para-aortic LNs, the percentages of cDCs were not significantly different between the solvent- and IFN-γ-treated groups, although IFN-γ still significantly reduced the proportions of pDCs. As mentioned above, pDCs are inherently more efficient at Treg induction; thus, we determined whether the proportions of Treg cells were changed following the imbalance between cDCs and pDCs induced by IFN-γ. As shown in Fig. 4d,e, the percentages of Treg cells were slightly lower in the spleens, but were significantly downregulated in the para-aortic LNs compared to the control. These results suggest that the expansion of Treg cells in the para-aortic LNs is more dependent on pDCs, which indicates a unique regulatory mechanism underlying pregnancy-related immunity tolerance.

Next, we evaluated the percentages of cDCs and pDCs in the decidua and myometrium on E8.5 with solvent- or IFN-γ-injected mice. Surprisingly, IFN-γ induced a significant reduction in pDCs (Fig. 4f); Treg cells also correspondingly decreased significantly in the decidua (Fig. 4g). However, the proportions of cDCs in both the decidua and the myometrium showed an upward trend, and the proportions of pDCs declined in the myometrium of the IFN-γ-treated mice, but the difference was not significant. These findings showed that pDCs may facilitate the development of Treg cells at the maternal-fetal interface, but are not the decisive factor for the elevated proportions of Treg cells in the decidua. Furthermore, we also studied the expression of IDO and MHCII in cDCs and pDCs, and little change in expression was found in the spleens and para-aortic LNs between solvent- and IFN-γ-treated mice. However, elevated expression of IDO and MHCII, indicating more intense alternative activation, was observed in both of the DC subsets in the para-aortic LNs (Fig. 4h).

### IFN-γ-augmented GM-CSF expression is supposed to disrupt the balance between cDCs and pDCs during pregnancy

**Figure 5:**
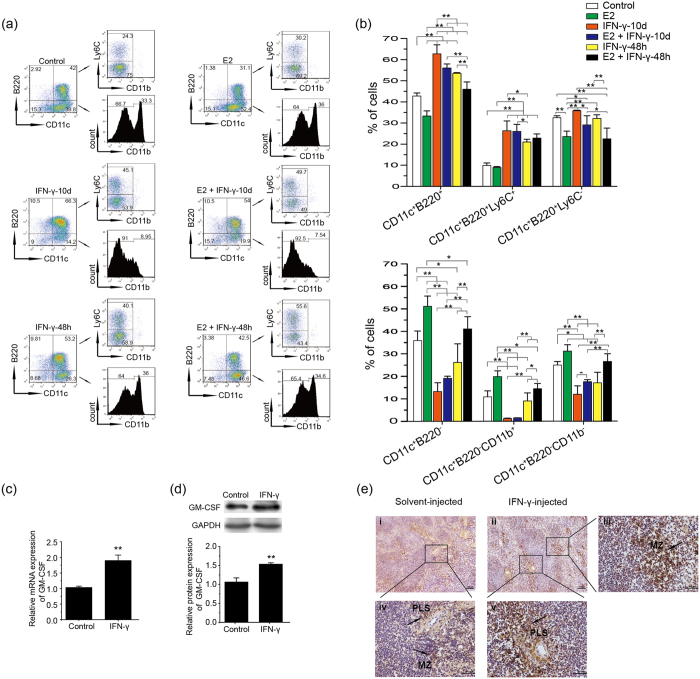
IFN-γ elevates GM-CSF to disrupt the balance between cDCs and pDCs. (**a**) Total BMs from female C57BL/6 mice were cultured in hormone-deficient medium with 100 ng/ml Flt3L with different treatments (10 nM E2; 300 U/ml IFN-γ). E2 was added at the starting point of the culture with all treatments. IFN-γ was added on D0 or last 48 h during the culture, respectively, and the cells were collected for analysis on D10. The percentages of cDCs (CD11c^+^ B220^−^), mDCs (CD11c^+^ B220^−^ CD11b^+^), LDCs (CD11c^+^ B220^−^ CD11b^− to lo^), pDCs (CD11c^+^ B220^+^), functional pDCs (CD11c^+^ B220^+^ Ly6C^+^) and unknown-function pDCs (CD11c^+^ B220^+^ Ly6C^−^) are shown for different treatments. (**b**) The histogram displays the statistical results of three independently repeated experiments. represent the mean ± SD. *P < 0.05; **P < 0.01. (**c,d**) GM-CSF expression of spleen cells was analyzed by quantitative PCR (**c**) and Western blotting (**d**) in three independently repeated experiments. The data represents mean ± SD. *P < 0.05; **P < 0.01. (**e**) GM-CSF expression in the spleen was analyzed by immunohistochemistry. Positive staining cells are indicated by arrows in the PLS and MZ of the spleens. Photomicrographs are representative of four mice in the control and IFN-γ-treated groups. Panels iii, iv and v are higher magnifications of black rectangle-marked areas. Scale bars: 100 μm (i and ii) and 50 μm (iii, iv and v).

Treg cells have been confirmed to be expanded systematically^34^ in an estrogen-dependent manner^35^. Additionally, estradiol (E2) modulates DC differentiation in different ways via GM-CSF- and Flt3L^36^, and type I IFNs can act synergistically with Flt3L to promote pDC development^26^. However, it still remains unclear whether estradiol can expand pDCs just like Treg cells during pregnancy and cooperate with IFN-γ to determine the differentiation rate of cDCs and pDCs during Flt3L-driven development. Therefore, we cultured total Bone Marrow cells (BMs) from female mice in hormone-deficient medium with Flt3L plus E2, IFN-γ or both. After 10 days, the proportions of cDCs and pDCs were examined using flow cytometry. The cDCs (CD11c^+^ B220^−^) were subdivided into myeloid DCs (CD11c^+^ B220^−^CD11b^+^) (MDC) and lymphoid DCs (CD11c^+^ B220^−^CD11b^− to lo^) (LDC)^36^, while the pDCs (CD11c^+^ B220^+^) were divided into Ly6C^+^ (functional pDC) and Ly6C^−^ subsets^37^. As shown in Fig. 5a,b, E2 promoted the differentiation of both MDCs and LDCs significantly while decreased Ly6C^−^ pDC proportions compared with the control group, but no change occurred in the percentage of functional pDCs. Furthermore, the addition of IFN-γ at the initiation of the culture can promote the differentiation of functional pDCs both in the absence and presence of E2, while the proportions of cDCs and especially MDCs were reduced remarkably. Even supplementation of IFN-γ for the last 48 h culture could still expand the functional pDC proportion significantly and reverse the effect of E2 in the differentiation of cDCs. The data suggest that IFN-γ can promote the Flt3L-driven development of pDCs, which is in contrast to the results obtained *in vivo*, thereby allowing us to explore the mechanism underlying the reduction in pDCs with elevated IFN-γ *in vivo*.

CCR9^−^ pDCs, present in the spleens and LNs as an immediate pDC precursor, spontaneously develop into CCR9^+^ pDCs, which can be diverted from the pDC lineage and differentiate into cDCs under the influence of GM-CSF^38^. Hence, we investigated whether elevated IFN-γ can upregulate GM-CSF expression *in vivo*. As shown in Fig. 5c,d, the mRNA and protein levels of GM-CSF were both significantly enhanced in the spleens of IFN-γ-treated mice. Furthermore, histological analysis showed stronger staining in the periarterial lymphatic sheath (PLS) and marginal zone (MZ), containing massive quantities of T cells or macrophagocytes, respectively, in the spleens of IFN-γ-injected mice compared with solvent-injected mice (Fig. 5e). Overall, our results suggest that IFN-γ can induce a marked increase in GM-CSF that may alter the differentiation fate of DC subsets.

## Discussion

Many gestational complications have been found to be related to elevated IFN-γ levels^39^. In this study, we show that IFN-γ can break the balance of cDCs and pDCs both in the peripheral immune organs and at the maternal-fetal interface during abnormal pregnancy with fetal resorption. To our knowledge, this is the first study indicating that the deleterious effects of elevated IFN-γ correlate with a change in the differentiation of cDCs and pDCs to affect immune tolerance.

As a particular DC subset, pDCs play a dominant role in mediating allograft tolerance when present in the secondary lymphoid organs, whereas they mediate allograft rejection in spleens^33^. The expansions of DCs and Treg cells are highly correlated in the para-aortic LNs, while no significant expansion of pDCs occurs in the spleen (Fig. 1b). In allogeneic mating, the fetus is usually regarded as an “alloantigen” that can expand Treg cells during pregnancy^40^. Thus, our results provide an important indication that pDCs may induce the expansion of antigen-specific Treg cells in secondary LNs to mediate tolerance; further studies are now being conducted in our lab. The accumulation of alloantigen-specific Treg cells is enhanced in the gravid uterus^41^. However, there are two major myeloid DC subsets with CD11b^lo^ CD103^+^ or CD11b^hi^ CD103^−^ surface phenotypes^42^, and the DC subset responsible for this process remains unknown, although uterine DCs (uDCs) have been shown as a whole to participate in immune tolerance induction by promoting the differentiation of Treg cells^43^. In the present study, we confirmed lower frequencies of both cDCs and pDCs present in the decidua compared to the myometrium, with no significant difference between the subsets, while a significantly higher frequency of Treg cells existed in the decidua; on the other hand, PDCs were significantly downregulated along with a decrease in the proportion of Treg cells in the decidua of IFN-γ-treated mice. The data indicate that pDCs at the maternal-fetal interface may facilitate tolerance by inducing Treg cell differentiation, but this is not a decisive mechanism underlying the Treg cells in the decidua compared to the myometrium. We first present a hypothesis that the proportions of cDCs and pDCs are regulated precisely during pregnancy and that breaking this balance will lead to abnormal pregnancy with fetal loss. However, it remains to be verified whether this represents a novel indicator for human spontaneous abortion in clinical practice.

The balance of Th1/Th2 cytokines is a well-known paradigm in building immune tolerance during successful pregnancy^44^, and IFN-γ, as a typical Th1 cytokine, is significantly augmented in patients with preeclampsia^29^ or unexplainable recurrent spontaneous abortion^30^, indicating that a supraphysiological level of IFN-γ is harmful to normal pregnancy. In our lab, abortion can be induced in mice injected with 5000 U of IFN-γ only once on D6^31^, and IFN-γ-treated (Fig. 4a) or Treg cell-deleted gravid mice^34^ exhibit a similar phenotype of fetal absorption. In this study, we first demonstrated that elevated IFN-γ can disrupt the balance between cDCs and pDCs to damage tolerance by reducing the development of pDC and downregulating Treg cells in the para-aortic LNs and decidua. These results further suggest that pDCs are critical for inducing alloantigen-specific tolerance during pregnancy and deepen our understanding of IFN-γ, which can modulate the frequencies of immune cells to influence the outcome of pregnancy.

CCR9^−^ pDC–like precursors, found in many peripheral lymphoid and nonlymphoid organs, tend to differentiate into CCR9^+^ pDCs in the bone marrow and liver, while they give rise to cDCs in peripheral lymphoid organs^45^. The factors that dominate tissue-specific differentiation decisions remain unclear. Hence, the change in the proportion of cDCs and pDCs in the para-aortic LNs during pregnancy progression (Fig. 1b) will be an ideal model to explore the regulatory mechanism underlying the differentiation of CCR9^−^ pDC–like precursors in the steady state. GM-CSF can prevent pDC development and induce the differentiation of CCR9^−^ pDC–like precursors into cDCs *in vitro*^38^. In the present study, we further indicated that IFN-γ may promote GM-CSF expression to block pDC differentiation and increase cDC proportions to destroy their balance *in vivo*. GM-CSF has been proved to have divergent effects on the outcome of pregnancy. The mice treated with a lower dose of GM-CSF showed reduced fetal resorption rates, while a higher dose of GM-CSF sometimes, but not always, enhanced the frequency of fetal resorptions^46^. GM-CSF-educated DCs can prevent the occurrence of abortions^47^,^48^, and low-dose GM-CSF promotes the development of OX40L^+^ Jag^+^ DCs, which may be responsible for maintaining Treg homeostasis *in vivo*^49^. Furthermore, GM-CSF can modulate DC differentiation depending on its dose and the presence of other cytokines to play a pro –inflammatory or regulatory role^49^. Therefore, it deserves to be studied further whether GM-CSF alone or together with other cytokines such as IFN-γ can change the differentiation of cDCs and pDCs to result in pregnancy failure.

In summary, the balance between cDCs and pDCs is pivotal for a successful pregnancy, and IFN-γ may break this balance and lead to pregnancy failure. Therefore, we provide a novel target that should be taken into consideration for the prevention of human spontaneous abortion in the future.

## Methods

### Mice

Eight-week-old female C57BL/6 and 10-week-old male BALB/c mice were purchased from the Experimental Animal Center of the Chinese Academy of Medical Sciences, and OTII mice were purchased from Jackson Laboratories. The mice were housed in a temperature- and humidity-controlled room with a constant 12 L/12D photoperiod and were able to eat and to drink water ad libitum. Studies performed with the mice were approved by the Institutional Animal Care and Use Committee of the Institute of Zoology, Chinese Academy of Sciences. All animal experiments were conducted in accordance with Institutional Animal Care and Use Committee guidelines. Allogeneic mating was achieved by caging female C57BL/6 mice with male BALB/c mice at a 2:1 ratio, and the first day with the presence of a vagina plug was validated as pregnancy day 0.5 (E0.5).

### IFN-γ-induced abortion model

Allogeneic mated female C57BL/6 mice were injected with 5000 U IFN-γ (PeproTech) or placebo i.p. from E5.5 to E7.5, once a day. Then, the mice were killed by cervical dislocation on E8.5, and fetal resorption was observed as a marker of abortion.

### Cell suspension preparation

Spleens and para-aortic LNs were separated from placebo-treated or IFN-γ-induced-abortion female mice and gently mechanically dispersed. The erythrocytes were lysed using an ammonium chloride lysis solution. Then, the cells were washed and resuspended in 0.1% BSA (PBS) for further staining.

The deciduas/myometrium on E8.5 and the plancenta/myometrium on E13.5 were isolated and minced into small fragments. Minced tissues were placed in HBSS containing 200 U/ml hyaluronidase, 1 mg/ml collagenase type IV, and 0.2 mg/ml DNase (Sigma-Aldrich) for 40 min at 37 °C. After the digestion, the cells were washed and incubated for another 15 min in 0.1% BSA (PBS) at room temperature before filtration through a 37 μm nylon mesh. Then, the cells were collected and resuspended for further staining.

### Surface and intracellular staining and flow cytometry

Cells were resuspended and incubated with CD16/CD32 blocking antibodies for 20 min, then with labeled antibodies for 30 min at 4 °C. The antibodies used, listed as follows, were purchased from eBioscience or BioLegend: plycoerythrin (PE)-conjugated anti-CD11c (clone N418), fluorescein isothiocyanate (FITC)-conjugated anti-CD11b (clone M1/70), eVolve 605-conjugated anti-CD11b (clone M1/70), eFluor 450-conjugated anti-Ly6C (clone HK1.4), FITC-conjugated anti-B220 (clone RA3-6B2), PE-Cyanine7-conjugated anti-MHCII (clone M5/114.15.2), peridinin chlorophyll protein (PerCP) Cyanine5.5-conjugated anti-CD45 (clone 30-f11), allophycocyanin (APC)-conjugated anti-PDCA-1 (clone 927), FITC-conjugated anti-CD4 (clone GK1.5), PE-conjugated anti-CD25 (clone PC61.5). After surface staining, the cells were rinsed with PBS containing 0.1% BSA for analysis. For intracellular antigen staining, the Intracellular Fixation & Permeabilization Buffer Set (eBioscience) was used according to the manufacturer’s protocol, then the cells were intracellularly stained with PerCP Cyanine5.5-conjugated anti-IDO (clone mIDO-48); the Foxp3/Transcription Factor Staining Buffer Set (eBioscience) was used, and then the cells were stained APC-conjugated anti-FoxP3 (clone FJK-16 s). The samples were analyzed on a Becton Dickinson FACSCalibur or FACSAria III.

### Inducing DC differentiation with Flt3L

Femurs and tibias were used to isolate BMs from female mice. The cells were cultured in steroid hormone-deficient medium with phenol red-free RPMI 1640 (Corning), 10% charcoal dextran-stripped FBS (Biolnd) containing Flt3L (100 ng/ml, PeproTech) plus E2, IFN-γ or both to induce DC differentiation for 10 days or IFN-γ for last 48 h during the culture; then, the cells were collected for further analysis.

### The isolation of cDCs and pDCs and the differentiation of Treg cells

To induce Treg cells, cDCs and pDCs were aseptically isolated from para-aortic LNs of female C57BL/6 gravid mice by flow cytometry, and CD4^+^ CD25^−^ T cells were purified from the spleen cells of OTII mice using a CD4^+^ CD25^+^ regulatory T cell Isolation Kit (Miltenyi Biotec). Different DC subsets (1 × 10^4^) were incubated with 1 μg/ml OVA peptide (ISQVHAAHAEINEAGR) before co-culturing with T cells (1 × 10^5^) in 200 ml RPMI 1640 supplemented with 10% FBS (Gibco) for 7 days; then, the proportion of Treg cells was examined by flow cytometry.

### Real-time PCR

Total RNA was extracted using BioTeke kits and used as a template for reverse transcription using the Superscript First-Strand Synthesis System (Invitrogen). CDNA was used for quantitative real-time PCR with SYBR Green Master Mix (Com Win Biotech Co. Ltd). The primers used were the following: GM-CSF, Forward: 5′-GAAGATATTCGAGCAGGGTC-3′; Reverse: 5′-CATTACGCAGGCACAAAA-3′; GAPDH, Forward: 5′-AACTTTGGCATTGTGGAAGG-3′; Reverse: 5′-GGATGCAGGGATGATGTTCT -3′. PCR analysis was performed with a LightCycler 480 (Roche).

### Western blotting

Proteins were extracted from the spleens using lysis buffer (Applygen) plus protease inhibitors and phosphatase inhibitors (Roche), then subjected to SDS-PAGE followed by transfer to nitrocellulose membranes (Pall) and probing with GM-CSF (Boster) and GAPDH primary antibodies (Santa Cruz). The specific bands were visualized with an ECL Detection Kit (Pierce), and images were acquired using Kodak X-Omat film.

### Immunohistochemical staining

Cryosections of mouse spleens were fixed in 4% PFA for 10 min. After washing, the sections were treated with 3% H_2_O_2_ for 10 min at 37 °C and blocked in 5% bovine serum albumin (Invitrogen) for 1 h sequentially. Then, the sections were incubated with anti-GM-CSF primary antibody (eBioscience) overnight at 4 °C, then subsequently incubated with an HRP-conjugated secondary antibody at 37 °C for 2 h. Finally, the slides were stained with diaminobenzidine and counterstained with hematoxylin; then, the slides were photographed using a Nikon H660L microscope.

### Statistical analyses

All statistical analyses were performed using analysis of variance (ANOVA) in SPSS version 20.0. P values < 0.05 were defined as statistically significant.

## Additional Information

**How to cite this article**: Fang, W.- *et al.* The Balance between Conventional DCs and Plasmacytoid DCs Is Pivotal for Immunological Tolerance during Pregnancy in the Mouse. *Sci. Rep.* **6**, 26984; doi: 10.1038/srep26984 (2016).
